# Chromatin protein PC4 is downregulated in breast cancer to promote disease progression: Implications of miR-29a

**DOI:** 10.18632/oncotarget.27325

**Published:** 2019-12-03

**Authors:** Sweta Sikder, Sujata Kumari, Manoj Kumar, Shrinka Sen, Namrata Bora Singhal, Srikumar Chellappan, Mukul Godbole, Pratik Chandrani, Amit Dutt, Kodaganur S. Gopinath, Tapas K. Kundu

**Affiliations:** ^1^Jawaharlal Nehru Centre for Advanced Scientific Research, Bangalore, India; ^2^H. Lee Moffitt Cancer Center and Research Institute, Tampa, Florida, USA; ^3^Integrated Cancer Genomics Lab, Advanced Centre for Treatment, Research and Education in Cancer, Mumbai, India; ^4^Bangalore Institute of Oncology, Bangalore, India

**Keywords:** tumour suppressor, non-histone chromatin protein, oncomiR, matrix metalloproteinases, p53 activation

## Abstract

The human transcriptional coactivator PC4 has numerous roles to play in the cell. Other than its transcriptional coactivation function, it facilitates chromatin organization, DNA damage repair, viral DNA replication, etc. Although it was found to be an essential protein *in vivo*, the importance of this multifunctional protein in the regulation of different cellular pathways has not been investigated in details, particularly in oncogenesis. In this study, PC4 downregulation was observed in a significant proportion of mammary tissues obtained from Breast cancer patient samples as well as in a subset of highly invasive and metastatic Breast cancer patient-derived cell lines. We have identified a miRNA, miR-29a which potentially reduce the expression of PC4 both in RNA and protein level. This miR-29a was found to be indeed overexpressed in a substantial number of Breast cancer patient samples and cell lines as well, suggesting one of the key mechanisms of PC4 downregulation. Stable Knockdown of PC4 in MCF7 cells induced its migratory as well as invasive properties. Furthermore, in an orthotopic breast cancer mice model system; we have shown that reduced expression of PC4 enhances the tumorigenic potential substantially. Absence of PC4 led to the upregulation of several genes involved in Epithelial to Mesenchymal Transition (EMT), indicating the possible mechanism of uniform tumour progression in the orthotropic mice. Collectively these data establish the role of PC4 in tumour suppression.

## INTRODUCTION

The eukaryotic cell employs several infallible mechanisms to safeguard its genome. Systematic organization of the chromatin with the help of histones and non-histone proteins provide the first armour to the genetic material. Cells maintain their genomic integrity through modulation of the chromatin by various factors, or through the regulation of catabolic pathways like autophagy, and DNA repair pathways. Disruption of these regulated safeguard mechanisms might result in genomic instability and cause tumorigenesis. The genome of tumor cells is highly unstable. The genomic instability confers advantages to the highly proliferating tumor cell population by shortening of cell cycle and/or bypassing various intracellular and immunological control systems. Studies have revealed that chromatin structure is one of the key determinants of somatic mutation rates in cancer cells [1]. Disruption of epigenetic language also has been shown to correlate with the occurrence of cancers [2]. Moreover, the imbalance of histone modifications causing establishment of altered chromatin structure can critically affect the transcriptome of the cell pushing it towards carcinogenesis. To thrive, cancer cells induce changes in the cytoskeleton network, cell to cell adhesion property, and events like EMT resulting in its propagation. Apart from this cancer cells also exhibit better cell survival against barriers posed by tissue microenvironment and external stimuli. The chromatin-associated proteins (CAPs) like High Mobility Group family of proteins (HMGs), linker histones, proteins belonging to heterochromatin protein family, MeCP2, PARP etc., which dynamically interact with basic histone-DNA filaments, fine-tune the genome organization and thereby gene functions [3]. Diverse kinds of CAPs lead to varied functional outcome based on their mode of interaction with different chromatin components. These proteins and other chromatin proteins suffer alteration during cancer in various ways namely in their mode of expression, localization, and mutations etc.; affecting different pathways [4]. Among architectural proteins, a notable example is the overexpression of HMGA which represents the hallmark of several malignancies and benign tumours [5,6]. Extensive studies on human heterochromatin protein 1 (HP1) isoforms in cancer show that it regulates androgen receptor signalling and enhances cell growth in prostate cancer. However, the expression of HP1α was found to be biphasic during breast cancer progression [7]. Presently, the mechanistic contributions of most of these proteins towards cancer manifestation are poorly understood.

PC4, also known as SUB1, was discovered as a transcriptional co-activator of activator dependent RNA polymerase II driven transcription [8–10]. In accordance with this function, PC4 is found to enhance DNA binding ability of p53 and thereby its tumour suppressive functions [11,12]. Interestingly, PC4 was also found to be a p53 responsive gene [13]. Apart from its transcriptional coactivation function, PC4 was found to be a bonafide component of the chromatin involved in the chromatin organization (compaction) through direct interaction with the histones [14]. Absence of PC4 leads to a dramatic alteration of chromatin organization and epigenetic state [15]. While PC4 is known to regulate chromatin templated organization as well as function, it is largely unknown how it could influence cancer manifestation. It has been shown that PC4 interacts with AP2-α through its C-terminal domain and inhibits AP2 transcriptional self-interference [16]. PC4 was also found to be a potent activator of p53 suggesting it’s *in vivo* role as a tumour suppressor. However, no study has conclusively reported such a role for PC4 till date. On the contrary, the expression of PC4 was found elevated in most types of cancer like prostate, astrocytoma, [17–20]. Owing to the complex roles of PC4 in the maintenance of genome, we explored the role of PC4 in breast cancer manifestation. In the present study, we examined the PC4 expression profile in breast cancer patient samples where, majority of the samples showed a significant downregulation both at the protein and transcript levels, regardless of their receptor type. We have identified miR-29a as a regulator of PC4 expression. It was found that indeed in several patient samples and patient sample derived highly metastatic cell lines, the miR-29a was upregulated. Downregulation of PC4 in Breast cancer cell lines was found to induce the tumourigenicity. In agreement with this observation in an orthotopic breast cancer mice model system, knockdown of PC4 leads to better tumor progression. These data strongly argue to establish PC4 as a tumour suppressor.

## RESULTS

### PC4 expression gets downregulated in breast cancer

**Figure 1:**
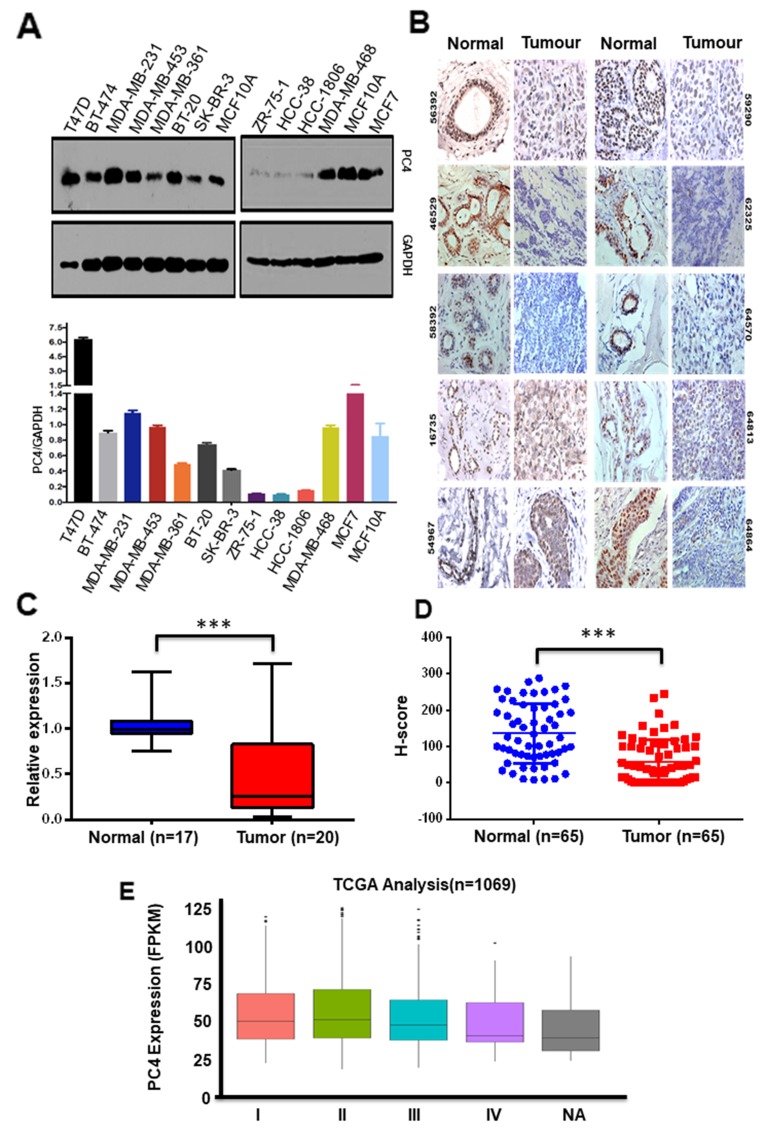
PC4 expression is predominantly downregulated in breast cancer patient samples **(A)** Western Blot analysis of PC4 was done with lysates prepared from different breast cancer cell lines. GAPDH expression was used as the loading control. The lower panel depicts a bar graph for densitometric analyses of protein bands which were done by considering the raw intensity values of the bands of PC4 and GAPDH as obtained from ImageJ analysis. The ratios of PC4/GAPDH for each cell line were then compared against MCF-10A. n=2 **(B)** Representative images of IHC performed on breast cancer and adjacent normal tissue sections from the same patient with highly purified and characterized polyclonal anti- PC4 antibodies; imaged at 40x magnification. The number written on the left top of the images indicates patient ID. Patient samples represent an Indian cohort obtained from Bangalore Institute of Oncology/HCG, Bangalore **(C)** PC4 transcript analysis from Breast cancer tumour samples as compared to the adjacent normal. PC4 expression was checked with gene-specific primers. The fold change is calculated by the ∆∆Ct method (see Materials and methods). Actin specific gene primers were used for normalization. n represents the number of patient samples used for the analysis. **(D)** Comparative analysis of PC4 expression as obtained from IHC data of normal vs tumour of Breast cancer patient samples were represented as H-score. Data are presented as means ± S.E.M. p^*^ < 0.01, p^**^ < 0.001, p^***^ < 0.0001. **(E)** PC4 transcript analysis from 1069 Breast cancer patient samples obtained from TCGA database, stage-wise. The number of patient samples analyzed for each stage is mentioned in the supplementary section.

Multifunctional human chromatin-associated protein PC4 is an essential nuclear protein [22] performing various critical functions of the cell. Besides its role in transcription, PC4 plays important functions in genome stability [23,24] and cell seggregation [25]. Alteration of expression of this protein might lead to various physiological defects and probably pathogenesis. We, therefore, resorted to check its expression in Breast cancer where the genome instability plays a pivotal role in tumour progression. Breast cancer being a heterogeneous disease, the expression status of PC4 in different breast cancer cell lines exhibiting different molecular signatures, was studied. In a panel of thirteen breast cancer cell lines, having different origin and having different potential for invasion or migration property, the expression for PC4 was checked. Western blotting analysis across 13 breast cancer cell lines (Figure 1A) exhibiting varied molecular subtypes revealed significant downregulation of PC4 in 3 cell lines, as compared to the expression of MCF10A (normal epithelial breast cell line which is non-tumorigenic). PC4 was found to be substantially downregulated in protein level in the 3 highly aggressive cell lines ZR-75-1, HCC38, HCC1806. SK-BR-3 and MDA-MB-361 showed moderate downregulation in PC4 expression as compared to MCF7 cell lines. The other cell lines do not show any significant change while in T47D it was found to be upregulated. Breast cancer although regarded as a single disease varies considerably in their molecular signatures and gene expression, similarly the panel of Breast cancer cell lines reflects these variabilities and thereby the expression of PC4. It was interesting to note that despite the variation, PC4 was significantly downregulated in most of the cell lines exhibiting higher invasive and migratory property, and also the property of radiation resistance as in case of ZR-75-1. This signifies the potential role of PC4 in the process of Breast cancer oncogenesis. This encouraged us to look into the PC4 expression pattern in Breast cancer patient samples. Taking a cue from the observation obtained from the panel of Breast Cancer patient cell lines, we looked into the PC4 expression pattern from Breast cancer tumors obtained directly from patients. Immunohistochemistry analysis was carried out with a specific antibody against PC4 (Figure 1B). Adjacent normal tissues were taken as normal or control samples. Analysis of RNA from 20 pairs of Breast tumor tissues revealed suggestive downregulation of PC4 at the transcript level (Figure 1C). This was in correlation with the expression analysis of PC4 as observed in the aggressive and invasive breast cancer cell lines. However, in this IHC analysis (in 65 representative Indian patient samples), we find PC4 majorly downregulated in most of the tumor tissues (Figure 1D). The normal sections show an intense nuclear positivity in the epithelial cells lining the lactiferous ducts. However, the expression of PC4 was either found absent or very low in the cancer samples which could be easily made out by the rich haematoxylin (blue) counterstain. It is important to note that the normal tissue architecture was found to be disrupted in cancerous specimens and therefore sheets of cells with big nuclei are to be considered for comparison with the normal ductules. The cancer tissues belonging to Moffit Cancer Centre, USA also showed a similar trend of downregulation (Supplementary Figure 1A). The extent of nuclear immunoreactivity of anti-PC4 antibodies was further quantified employing H-score system. Statistical analysis performed with 50 samples from Moffit Cancer Centre, USA revealed ~45% reduction in PC4 expression (Supplementary Figure 1B and 1C) whereas, the Indian samples showed 62% reduction (Figure 1D). Correlation studies performed with Indian samples revealed an overall declining trend in the expression of PC4 with increasing tumor grade (data not shown). Similarly, a reduction trend was also seen with increasing age of Indian patients. The American patient samples did not show any significant correlation of PC4 expression with progressive stage or age of the patients (Supplementary Figure 1D). Similarly, we also investigated the expression of PC4 in two breast cancer subtypes (ER +ve and Triple-ve) using immunohistochemistry. A drastic downregulation in of PC4 was observed in both the cases as compared to the adjacent normal tissue (Supplementary Figure 1E). Thus downregulation of PC4 might not be affected due to the age, or the molecular signatures making it possibly a more universal phenomenon across different types of Breast Cancer. Analysis of PC4 expression in a large dataset of Breast cancer patient samples (1069) obtained from The Cancer Genome Atlas (TCGA database) was done (Supplementary Table 1). PC4 was found to be downregulated progressively across the different stages of Breast cancer (Figure 1E) fortifying its significant role in cancer progression.

### PC4 downregulation enhances the oncogenic properties of breast cancer cells

**Figure 2:**
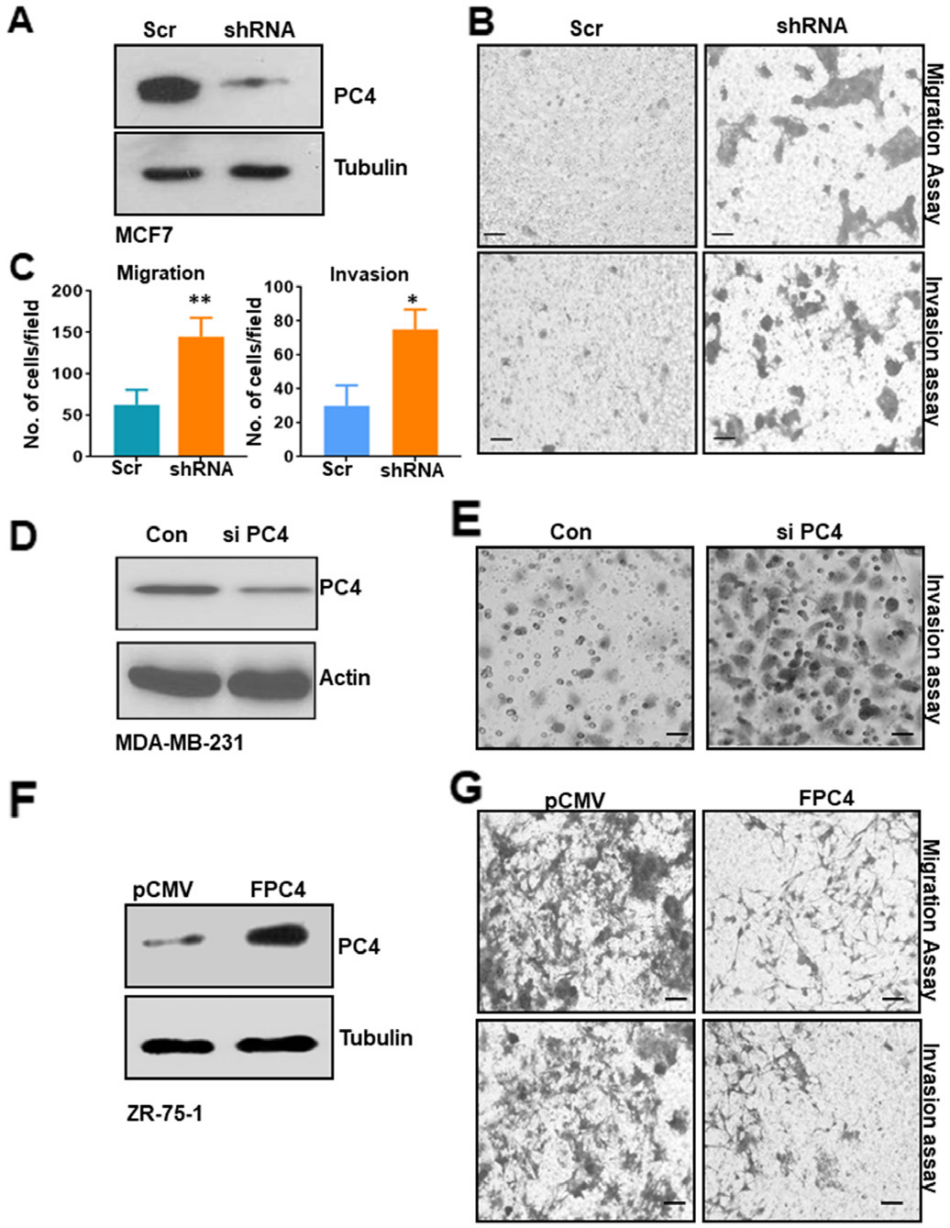
PC4 downregulation in breast cancer cell line enhances its tumorigenic properties **(A)** Western blot analysis for PC4 expression in non-silencing shRNA MCF7 cells and stable PC4 shRNA harbouring cells. Tubulin is used as a loading control. **(B)** An equal number of MCF7 cells harbouring stably the scrambled shRNA as well as the PC4 shRNA was seeded in the upper chamber of the Boyden Chamber assay with (for invasion assay, lower panel) or without matrigel (for migration assay upper panel). After 16hrs-24hours the cells in the lower chamber were stained with 10% Crystal Violet solution and then imaged for three independent fields. Scale bar represents 100μm **(C)** Left panel indicates a bar graph representing the quantitation of the migratory ability of the MCF7 PC4 knockdown cell lines compared against the scrambled control. Images of 3 independent fields from 2 biological replicates were used for quantitation. Similarly, the right panel indicates a bar graph which represents the quantitation of the invasive ability of the MCF7 PC4 knockdown cell lines compared against the scrambled control. Image of 3 independent fields from 2 biological replicates was used for quantitation. Data are presented as means ± S.E.M. p^*^ < 0.01, p^**^ < 0.001, p^***^ < 0.0001. **(D)** Silencing of PC4 using specific siRNA in MDA-MB-231 was confirmed by western blot analysis after control and PC4 siRNA transfection for 48 hours. Actin was used as a loading control. **(E)** Boyden chamber invasion assay performed in the above mentioned MDA-MB-231 cells. **(F)** Restoration of PC4 expression in ZR-75-1 cells inhibits its migratory and invasive property. Flag PC4 was transiently expressed in ZR-75-1 Breast Cancer cells and the expression was checked after 48hrs post-transfection by immunoblotting with PC4 antibody. **(G)** Equal number of ZR-75-1 cells transfected either with pCMV or FPC4 was seeded in the upper chamber of the Boyden Chamber assay with (for invasion) or without matrigel (for migration). After 16hrs-24hours the cells in the lower chamber were stained with 10% Crystal Violet solution and then imaged for three independent fields. Scale bar represents 100μm.

To understand the putative role of PC4 in Breast cancer progression, we wanted to analyse the significance of downregulation of PC4 in Breast Cancer cells. For this, a stable knockdown of PC4 in MCF7 cells was established (Figure 2A). Boyden chamber matrigel assay was carried out to measure the invasive property of the cells while the migratory property was assayed by monitoring the cells in the lower chamber of the Boyden transwell cup without the matrigel layer. Downregulation of PC4 led to higher migration of MCF7 cells (Figure 2B upper panel, Figure 2C). Cancer cells tend to invade through the basement membrane which is the first step in solid tumor metastasis. MCF7 cells harbouring sh-RNA against PC4 stably were also found to invade significantly the matrigel layer (Figure 2B lower panel, Figure 2C). Transient silencing of PC4 (Supplementary Figure 2A) was also done in three epithelial cell lines to support our observations in the stable knockdown cells. HBL-100 MCF7 and MDA-MB-231 cells were used in the order of increasing invasiveness. PC4 silenced cells migrated faster as compared to control (scramble siRNA) in both HBL-100 and MCF7cells (Supplementary Figure 2B).Breast cancer cell lines MDA-MB-231 and MCF7 were also subjected to Boyden chamber invasion assay upon transient PC4 silencing (Figure 2D and Supplementary Figure 2A). Breast cancer cell lines transfected with PC4 siRNAs could invade significantly more efficiently through matrigel than scrambled siRNA transfected cells (Figure 2E, Supplementary Figure 2C and 2D). Collectively, these data suggest that downregulation of PC4 harboured oncogenic properties to a lesser invasive Breast cancer cells (MCF7, HBl-100) and enhances the invasive properties of MDA-MB-231. This partially explains the putative tumour suppressor role of PC4 in Breast cancer progression. To validate the potential tumour-suppressive role of PC4 we further resorted to an approach of rescuing PC4 expression in the PC4 depleted Breast Cancer cells. For this, mammalian expression constructs of Flag-tagged PC4 was transiently transfected into ZR-75-1 cells and the expression of PC4 was checked by immunoblotting with anti-PC4 antibody (Figure 2F). Expression of PC4 drastically reduced the migratory as well as the invasive ability of the ZR-75-1 cells as is evidenced by the transwell migration assay after 48 hours of transfection of Flag PC4 constructs in ZR-75-1 cells (Figure 2G). These observations tend to establish that PC4 directly regulates the migratory and invasive property of highly metastatic breast cancer cells validating its possible tumor suppressive function.

### hsa-miR-29 regulates PC4 expression

**Figure 3:**
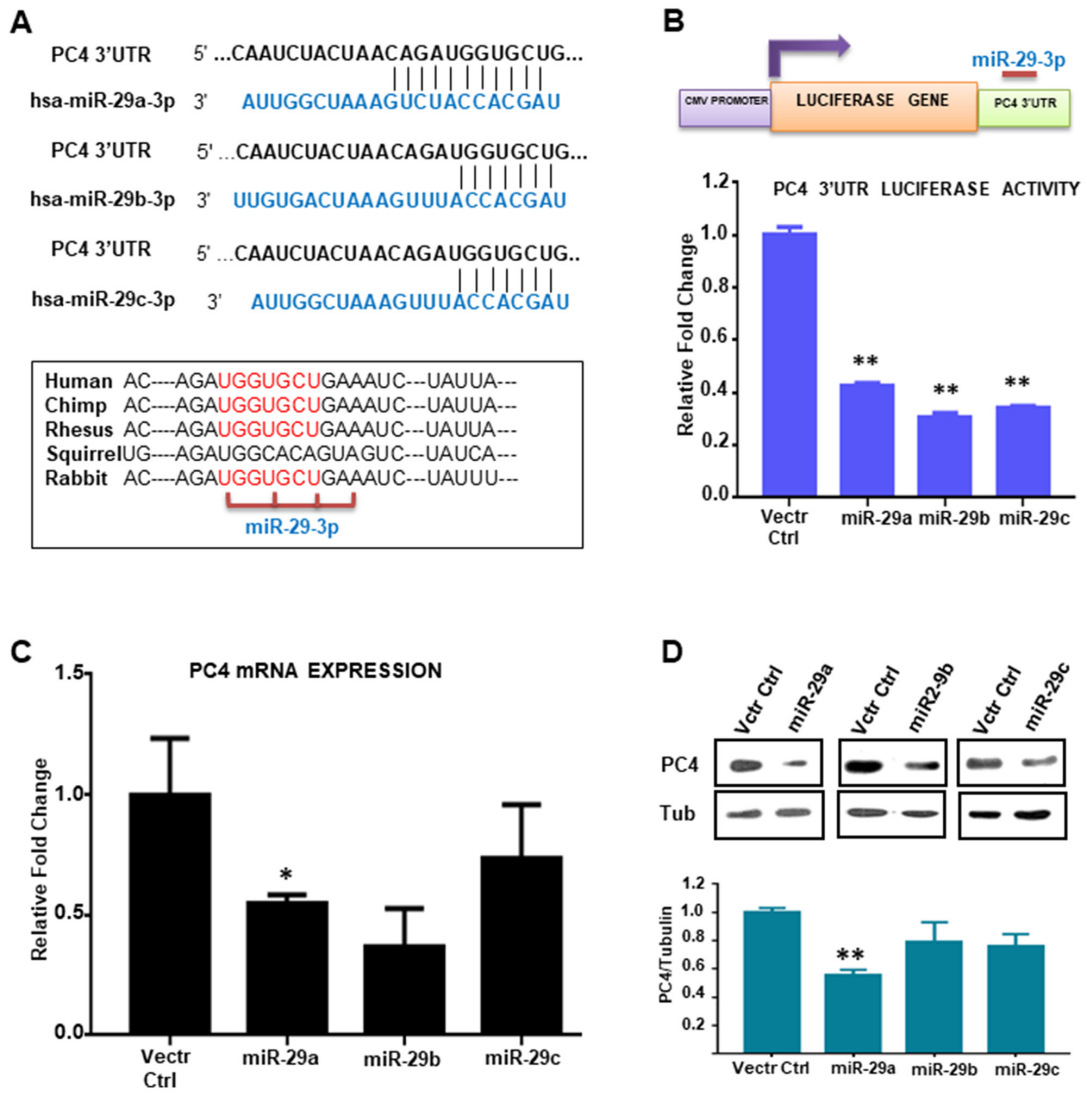
miR-29 targets and downregulates PC4 expression **(A)** Analysis of the 3'UTR of PC4 predicted the binding site of hsa-miR-29 family (29a, 29b, 29c) which is highly conserved across several species. **(B)** Regulation of PC4 3’UTR by miR-29. HEK293 cells were transiently transfected with the PC4 3'UTR expression construct along with pSUPER-miRNA expression plasmids. The 3'UTR region of PC4 is cloned into the MCS region pMIR-REPORT luciferase vector. Equivalent amount of control empty vector pSUPER was used to normalize the amount of transfected DNA. pMIR-βgal (500ng) was used as an internal control for all the transfection experiments to normalize luciferase activity. After normalization, relative fold change in luciferase activity was plotted (along y-axis). **(C)** PC4 expression was checked with gene-specific primers after transfection of HEK293 cells for 48hrs with miR29 expressing vector. Empty pSUPER vector was transfected as vector control. The fold change is calculated by the ΔΔCt method. Actin specific gene primers were used for normalization. **(D)** Ectopic expression of has-miR-29 downregulates PC4 expression in HEK293 cells. Western Blot analysis of PC4 was done after transfection with pSUPER empty vector (Vctr Ctrl) and miR-29 at 96 hours. Here Tubulin was used as the loading control. Densitometric analyses of protein bands were done and plotted as fold change (Lower panel).Data are presented as means ± S.E.M. p^*^ < 0.01, p^**^ < 0.001, p^***^ < 0.0001 (n=3, N=2).

The PC4 expression status in Breast Cancer patient samples encouraged us to investigate the factors regulating its expression, particularly in the cancer context. An earlier study on the regulator of expression of PC4 reveals that it is a p53 responsive gene [13]. Other factors regulating its expression are yet to be elucidated. PC4 is highly conserved across species. The PC4 gene is located on chromosome 5 which has five exons including one 5'-UTR (untranslated region). The 3'UTR region of PC4 was found to be almost 2kb long. Analysis of the 3'UTR region of PC4 by bioinformatics tools (Supplementary Figure 3) revealed several miRNA binding sites. The bioinformatics miRNA prediction tools reveal miR29 family to be one of the high scoring miRNAs which might regulate PC4 gene expression through its 3’UTR (Figure 3A and Supplementary Table 2).To experimentally validate the binding of miRNAs directly to the 3’UTR region of PC4, pMIR-REPORT luciferase vector containing the PC4 3’UTR sequence was employed. To address the binding propensity of miR-29 family (miR-29a, 29b and 29c) to the PC4 3’UTR, the miRNA expression constructs were co-transfected with the pMIR REPORT luciferase PC4 3’UTR plasmid in cells. The miR-29 family was found to repress the luciferase gene expression containing the PC4 3’UTR sequence (Figure 3B), thereby suggesting that it can bind to PC4 3’UTR and repress its expression. Taking cue from the 3’UTR luciferase-miRNA experiment we further investigated the effect of miR-29 family on PC4 expression at the transcript level. Pre miR-29a, miR-29b, miR-29c expressing plasmids were transiently transfected into HEK 293 cells for this purpose. After 48hours of transfection PC4 expression at the transcript level was checked. Overexpression of miR-29 significantly reduced expression of PC4 mRNA significantly (Figure 3C) thus suggesting the potential role of miR-29 as a negative regulator of PC4 expression. Western blotting analysis for PC4 expression at the protein level was also performed to ensure the alteration of endogenous PC4 level by overexpression of miR-29. Tubulin was taken as an endogenous control. Consistent with the luciferase as well as the RT-PCR data, overexpression of miRNAs in HEK293 cells showed a significant decrease in the endogenous level of PC4. (Figure 3D). Among all the miR-29 members miR-29a was found to be most effective to reduce the expression of PC4 (Figure 3D).

### hsa-miR-29a and PC4 expression is negatively correlated in breast cancer

**Figure 4:**
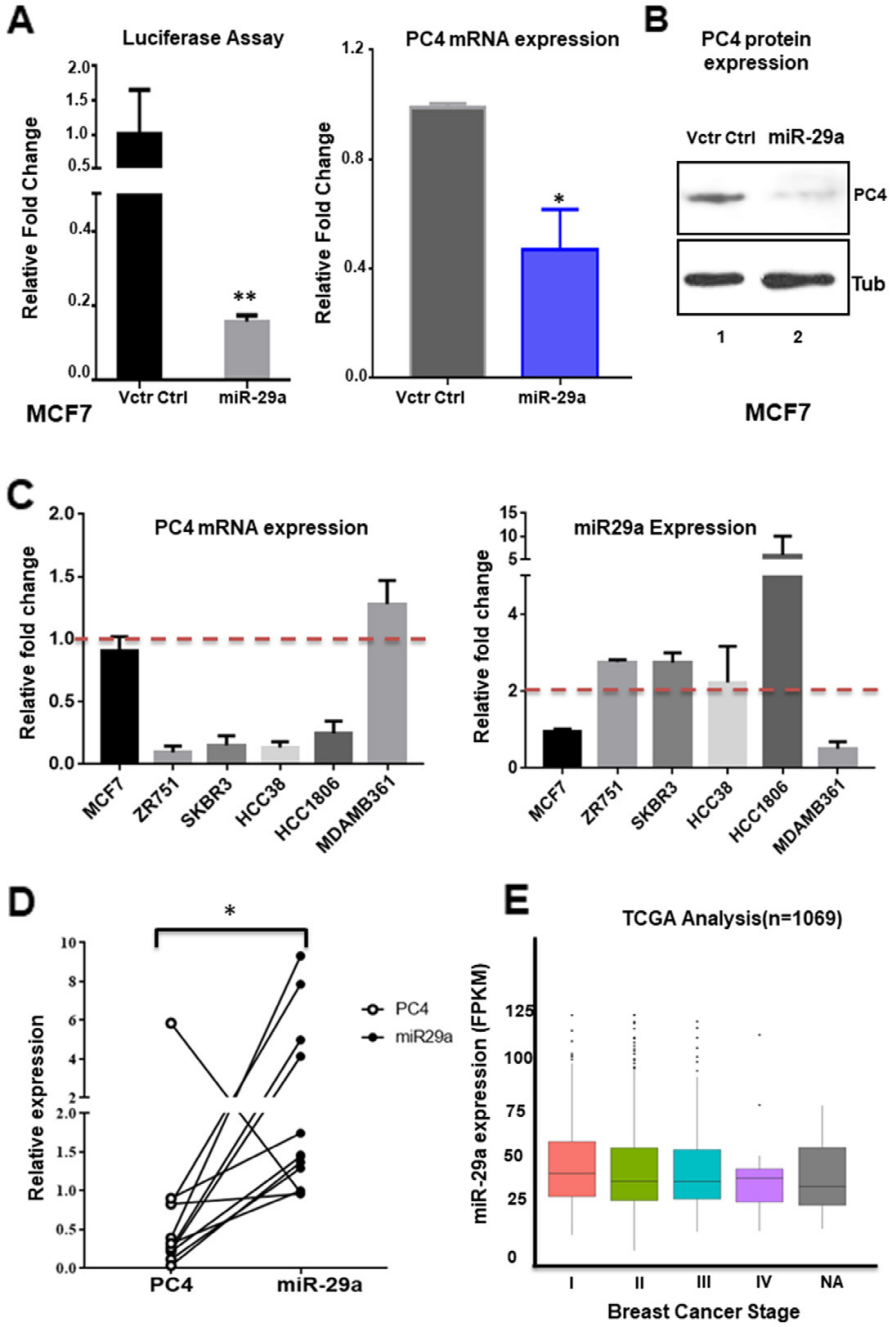
miR-29 modulates PC4 expression in Breast cancer cells **(A)** MCF7 cells were transiently transfected with the PC4 3’UTR expression construct along with miR-29a expression plasmid. Equivalent amount of control empty vector pSUPER was used to normalize the amount of transfected DNA. pMIR-βgal was used as an internal control for all the transfection experiments to normalize luciferase activity. After normalization, relative fold change in luciferase activity after 48 hours of transfection was plotted (along y-axis). PC4 expression was checked with gene-specific primers after transfection of MCF7 cells for 48hrs with miR-29a expressing vector. Empty pSUPER vector was transfected as vector control. The fold change is calculated by the ∆∆Ct method (see Materials and methods). Actin specific gene primers are used for normalization. **(B)** Western Blot analysis of PC4 was done from lysates obtained from MCF7 cells transfected with pSUPER empty vector (Vctr Ctrl) and miR-29a for 96 hours. Here Tubulin was used as the loading control. **(C)** Correlation of PC4 and miR-29a in Breast cancer cell lines. PC4 mRNA (left panel) and miR-29a (right panel) expression were checked with gene-specific primers in the panel of Breast cancer cell lines. The fold change is calculated by the ∆∆Ct method. Actin specific gene primers were used for normalization of mRNAs and U6 snRNA is used as a control for small RNAs. **(D)** PC4 and miR-29a expression were checked with gene-specific primers from RNA extracted from Breast cancer paired patient samples (normal vs adjacent tumour, n=15). The fold change is calculated by the ∆∆Ct method. Actin specific gene primers were used for normalization of mRNAs and U6 snRNA was used as a control for small RNAs. Data are presented as means ± S.E.M. p^*^ < 0.01, p^**^ < 0.001, p^***^ < 0.0001 **(E)** Transcript analysis of miR-29a (Expression) from 1069 Breast cancer patient samples obtained from TCGA database was analysed stage wise.

Our previous observations on the expression analysis of PC4 and miR29a prompted us to investigate the consequence of overexpression of miR29a in Breast cancer cells. Similar to the results obtained in HEK293 cells, we find that after 48 hours of miR29a overexpression in MCF7 cells results in decreased luciferase activity of PC4 3’UTR (Figure 4A). Hsa-miR-29a also led to the significant downregulation of PC4 mRNA (at 48 hours) (Figure 4A) as well as protein level when overexpressed in MCF7 cells (at 96 hours) (Figure 4B). This fortifies the role of miR-29a as a post-transcriptional regulator of PC4 expression in Breast cancer cells. To establish the potential role of miR-29a in downregulating PC4 expression in Breast Cancer we analysed the correlation of expression pattern of PC4 and miRNA-29a in Breast cancer cell lines or patient samples. We, therefore, checked the expression of both PC4 mRNA as well as miRNA in those breast cancer cell lines in which PC4 expression was found to be dramatically low at the protein level, as revealed by western blotting analysis. RT-PCR analysis shows that cell lines ZR-75-1, HCC-388, HCC-108, and SK-BR-3 possess low levels of PC4 mRNA as compared to that in MCF7 cells (Figure 4C left panel). Correspondingly, we also checked the levels of miRNA 29a in these cell lines. MiR-29a was found to be upregulated in all the cell lines where PC4 expression was found to be low (Figure 4C right panel). MDA-MB-361 and MCF7 were taken as control cell lines where PC4 expression was not found to be altered as compared to ZR-75-1/HCC-388. In agreement with this data, we find that miR-29a levels were either downregulated or unaltered in these cell lines. These data establish an inverse correlation of PC4 expression and that of miR-29a in the Breast cancer cell lines indicating that it could be acting as a regulator in the patient samples also. To further substantiate our hypothesis of miR-29a mediated downregulation of PC4 expression in Breast cancer patient samples as well; we carried out miRNA expression profiling of Breast Cancer patient samples. Among the 25 pair of Breast cancer patient samples analysed, we find there is indeed a negative correlation of PC4 expression and miR-29a expression for a subset of 12 samples (Figure 4D). This observation further validates that miR-29a might be one of the prime factors that might be responsible for the downregulation of PC4 both at transcript and protein level in Breast cancer patient samples. Although overexpression of miR29a in MCF7 cells significantly downregulated the expression of PC4 both at transcript and protein level, we note that the levels of PC4 expression in the highly invasive Breast cancer cell lines like ZR-75-1 and HCC-38 is not comparable to the former condition (the PC4 levels were further down in ZR-75-1 and HCC-38). Due to the existence of complex molecular subtypes of Breast Cancer, we hereby do not negate the role of other critical factors like repressors/DNA methylation which might be responsible for mediating the reduced expression of PC4 in Breast cancer patient samples as well as in the cell lines. Correlating the expression data of miR-29a in a large subset of Breast Cancer patient samples from TCGA database, we find that in stage IV miR-29a expression was relatively high than the other stages(Stage II and III) (Figure 4E). To a large extent, these data are in agreement with our PC4 expression analysis from the TCGA database where PC4 expression gradually decreased across different stages of Breast cancer the least being in Stage IV. Thus even at a larger subset of Breast cancer patient samples, we do find a possible correlation of miR-29a and PC4 expression validating miR29a as a negative regulator of PC4 expression and mediating its downregulation in Breast cancer patient samples.

### PC4 downregulation induces tumour progression *in vivo*

**Figure 5:**
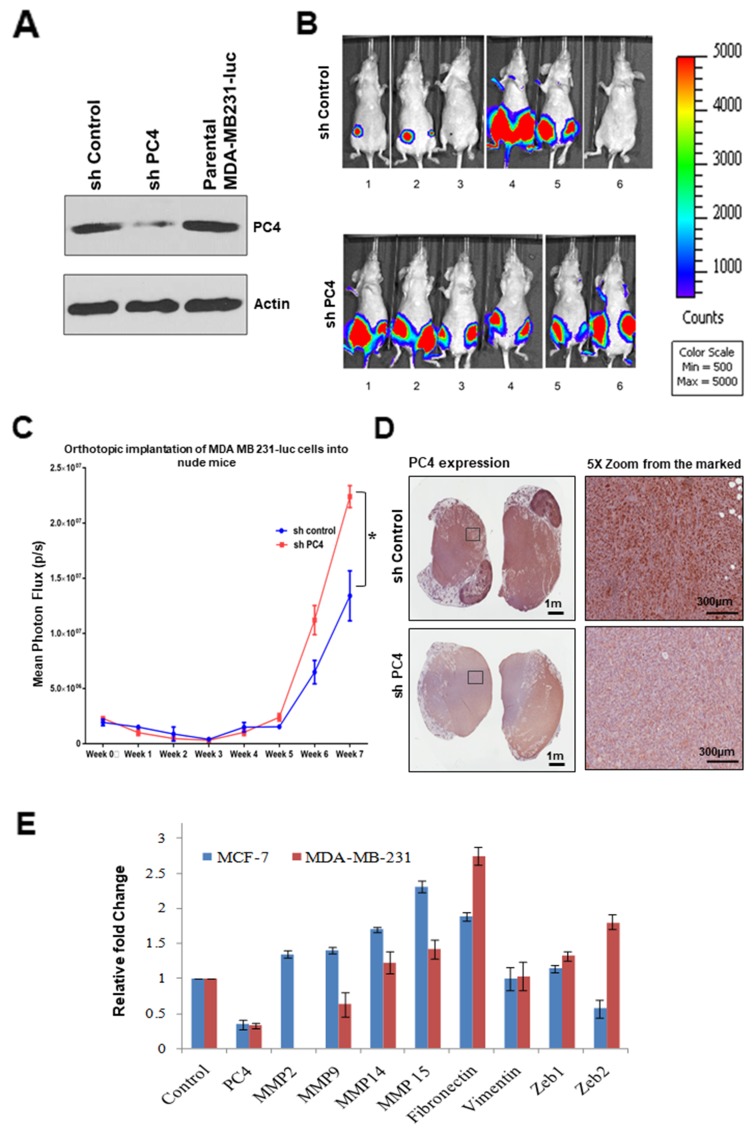
Downregulation of PC4 suppresses tumour progression in orthotopic breast cancer mice-model Absence of PC4 induces uniform tumour progression in orthotropic Breast cancer mice model. MDAMB231 cells harbouring an shRNA which targets PC4 was transfected and a stable knockdown cell line was established. (A) Right panel: Western blotting analysis to confirm PC4 knockdown in MDAMB231 luc cells was done using specific antibodies. (B) Left panel: Mice imaged for luc expressing cells after 9 weeks from the injection. Upper panel shows control mice, injected with MDAMB231 vector control cells which express PC4; lower panel represents a set of mice where PC4 was knocked down. **(C)** Assay of tumour progression by analysing the luminescence obtained from orthotropic implantation of MDA MB 231-luc cells (both control as well as sh-PC4) into nude mice for each week, until 8 weeks. p=0.015894952 **(D)** Representative images of IHC performed on tumour sections obtained from mice expressing control/sh-PC4 MDAMB-231 luc cells as shown in (A) to analyse PC4 expression. **(E)** Gene expression analysis upon PC4 silencing in Breast cancer cell lines. Real-time RT-PCR analysis of indicated transcripts upon transient silencing of PC4 in MCF-7 (Blue) and MDA-MB-231 (Red) cells are represented as bar graphs. Student’s t-test was performed for MMP14, MMP15 and fibronectin. p^*^ < 0.01, p^**^ < 0.001, p^***^ < 0.0001.

To reinforce the fact that PC4 might be acting as tumor suppressor in Breast Cancer, we carried out an orthotropic mice model-based experiments by employing MDA-MB-231 stable PC4 knockdown cells (Figure 5A). The PC4 knockdown cells were injected into the mammary fat pad tissue of mice and the tumor growth was monitored for 9 weeks. After 9 weeks, the group of mice injected with PC4 knockdown cells showed a uniform tumor progression as compared to the vector control cells (Figure 5B). Measurement of the mean luciferase counts from the tumours of each group of mice reveals that PC4 silencing led to uniform tumour formation significantly more than the control group (Figure 5C). This potentiates to the fact that PC4 indeed plays a tumour-suppressive role *in vivo*. Tumours obtained from both the groups of mice 9 weeks post-injection of MDA-MB-231 luc cells were analysed for PC4 expression. Immunostaining of tumour sections obtained from mice injected with MDA-MB-231 shPC4 luc cells show reduced PC4 expression (Figure 5D). This observation indicates that downregulation of PC4 favours tumour growth. *in vivo*. To understand the fundamental mechanism of PC4 downregulation related breast cancer tumour progression, we performed gene expression analysis in the MCF7 and MDA-MB-231 PC4 silenced cells. Since PC4 silencing could increase the migration and invasiveness in breast cancer cell lines, the expression pattern of different MMPs, mesenchymal markers like fibronectin, vimentin and regulators of EMT like, Zeb1 and Zeb2 was investigated upon silencing of PC4 in these cells. Interestingly, all the MMPs analysed and fibronectin showed higher expression when PC4 was downregulated in both the cell lines (Figure 5E). While MMP 14, MMP 15 and fibronectin showed marked up-regulation in both the cell lines Zeb1 and Zeb2 genes showed marginal up-regulation only in MDA-MB-231 cells upon PC4 silencing. Collectively these data suggest that PC4 could predominately have a repressive role on the overexpression of genes related to oncogenesis and metastasis.

## DISCUSSION

Global alteration of nuclear architecture as seen by loss of heterochromatin foci rendering nucleus more open in the absence of PC4, establishes it as one of the important chromatin architectural proteins. PC4 is essential for the maintenance of chromatin as its knockdown resulted in an accessible chromatin conformation as revealed by enhanced histone acetylation modifications [14,15]. Phenotypically, PC4 knockdown cell line demonstrated disruption of several cell cycle-related events at different levels [25]. Therefore, PC4 could be essential for guarding the genome against genotoxic insults and thereby prevent oncogenesis. The expression status of PC4 in Breast cancer patient samples correlated to our assumption of PC4 playing a tumour-suppressive role. A large dataset of Breast cancer patient samples showed significant downregulation of PC4 both at protein as well as transcript level irrespective of its molecular signatures signifying the universal tumour-suppressive role of PC4 across different subtypes of Breast cancer. This, however, is in contrast with the studies in most cancers including Breast where PC4 is found to be upregulated [17,18,26]. However, in our patient sample cohort both from India and USA, we find a significant downregulation of PC4 in coherence with the expression in highly metastatic Breast Cancer cell lines (Supplementary Table 3).

One of the most significant findings of the present study is the identification of a set of miRNAs which play a critical role in modulating PC4 expression even in the breast cancer patient samples. It is revealed that hsa-miR29 directly target PC4 3'UTR and thereby downregulate its expression. Furthermore the downregulation of PC4 mRNA level and also of the endogenous protein level upon overexpression of miRNAs (as validated by RT PCR data and western blot analysis) shows that PC4 might be a putative target of miR-29a. A recent study revealed that PC4 3’UTR region can bind to miR101, thereby getting downregulated particularly in the context of prostate cancer [18]. However in our bioinformatics prediction, this miRNA did not appear in the high scoring miRNA targets, and also miRNA 101 was reported to be downregulated in Breast cancer patient samples [27]. Our data suggest that there exists a distinct negative correlation of miRNA expression and PC4 expression in several Breast cancer patient samples and also in the cell lines where PC4 was found to be downregulated (Figure 4). Upon overexpression of miR29a in breast cancer cells, MCF7 in addition to the downregulation of PC4 expression, it also induces the migratory properties of the cells (data not shown). Stable knockdown of PC4 expression in MCF7 cells reduced dramatically its migratory and invasive property. This signifies further the potential role of PC4 in inhibiting Breast cancer tumorigenesis.ZR-75-1, breast cancer cells which harbour low levels of PC4 were found to be highly invasive. Restoring PC4 expression in this Breast cancer cell line depleted of PC4 reverted the property of invasiveness and migration. Collectively, all these data suggest the significance of PC4 in mediating oncogenesis and tumorigenic properties. To reinforce the fact that PC4 might be acting as tumor suppressor in Breast Cancer progression, we carried out an *in vivo* mice model study. In this orthotropic mice model system, MDAMB231 cells silenced for PC4 was injected into the mammary fat pad tissue of mice, and then the tumor growth was monitored and measured. After 9 weeks, the PC4 knockdown cells showed a uniform tumor progression as compared to the vector control cells (Figure 5B and 5C). This potentiates to the fact that PC4 indeed plays a tumour-suppressive role as its knockdown results in tumor formation and progression even in Breast Cancer *in vivo* mice model. Breast cancer cells devoid of PC4 show dysregulated expression of critical genes responsible for EMT pathways (Figure 5E). Our HEK293 PC4 knockdown data suggests that PC4 depletion alters the chromatin landscape through chromatin decompaction and thereby the epigenetic language of the cell [28]. Correlating with this, we find that in Breast cancer cells too, the genome is decompacted upon PC4 knockdown (data not shown). Upon mapping the open chromatin regions upon PC4 knockdown in HEK293 cells through ATACseq analysis, we also find substantial peaks critical regions adjacent to genes related to oncogenic pathways (data not shown here). Thus the altered transcriptome in the PC4 depleted Breast cancers cells might as well be due to the modified epigenetic and chromatin landscape which is now permissive to enhanced transcription.

Elucidating the expression pattern of chromatin-associated protein PC4 in the process of oncogenesis brings forth the significance of chromatin dynamics in cancer progression. The abnormal expression of the non-histone proteins, which are also involved in the maintenance of the chromatin structure, might result in these chromosomal abnormalities as well as the alteration in gene regulation which may drive oncogenesis. Recent advances in the field of epigenetics suggest that oncogenic development could be closely associated with the altered epigenetic state of the genome. Such epigenetic changes involve aberrant DNA methylation, the alteration of chromatin components in DNA packaging [2,29] and also an anomalous expression of noncoding RNAs like miRNAs. Microarray analysis from various tumor tissues has revealed the importance of miRNAs in the prediction, diagnosis and prognosis of tumor formation. Oncogenic miRNAs (oncomiRs) are usually overexpressed in cancers while tumour-suppressive miRNAs are downregulated quite similar to their mRNA counterparts. When these oncomiRs or tumor suppressor miRNAs are repressed or stimulated, respectively, the oncogenic properties of a tumor cell is significantly reduced [30]. Certain cancers become addicted to these oncomiR to such an extent that suppression of the oncomiR results incomplete reduction of the tumor [31,32]. However, it is intriguing to note that the miRNA expression pattern varies with cell tissue type as well as in various tumors. Our data suggest that although miR-29a could be a potential factor for the downregulation of PC4 in Breast Cancer there might be other cellular factors which either independently or in a concert affect its expression. This diversity of effects might be due to a large number of genes influenced by various regulatory chromatin-associated factors. Collectively, our data establish that the chromatin protein PC4 is a critical factor to suppress breast cancer progression.

## MATERIALS AND METHODS

### Immunohistochemistry

Breast cancer patient samples were collected at the Bangalore Institute of Oncology (BIO), India; and IHC were performed using standard protocols. H-score estimating the percentage of cells (0—100%) in each intensity category (0—3+) to calculate a final score, was given a range of 0 to 300. Similar IHC was performed in H. Lee Moffitt Cancer Center and Research Institute, Florida using human breast cancer tissue microarray slides; cat no. IMH-364, 371; Imgenex, and the H scoring for the same, was performed as described elsewhere. [21]

### Scratch assay

The scratch assays were performed using 80% confluent cells which were scratched with a sterile pipette tip in four separate places and image was captured after indicated time points. For experiments with Mitomycin-C, cells were treated with 10 mg/mL for 3 hours.

### Stable cell line generation

MCF7 cells constitutively expressing shRNA (Clone ID: V3LHS_331786, Dharmacon pGIPZ shRNA; Mature sequence: TTTTCTGGAGCAACTTGCT) against the ORF of PC4 mRNA was established to make a PC4 knockdown cell line. To negate the effect of shRNA transduction a cell line expressing a non-silencing shRNA control was also established. The non-silencing control hairpin sequence is as follows: 22mer sense: ATCTCGCTTGGGCGAGAGTAAG 22mer antisense: CTTACTCTCGCCCAAGCGAGAG This sequence does not match any known mammalian genes (had at least 3 or more mismatches against any gene as determined via nucleotide alignment/BLAST of 22mer sense sequence). These cell lines were generated using 10 μg pGIPZ lentiviral shRNAs targeting PC4 and helper plasmids (5 μg psPAX2, 1.5 μg pRSV-Rev, 3.5 μg pCMV-VSV-G). 10 μg of sh-plasmid was mixed with helper plasmids (5 μg psPAX2, 1.5 μg pRSV-Revs, 3.5 μg pCMV-VSV-G) and were co-transfected into HEK293T cells using the calcium phosphate method. 48 hours post-transfection media containing assembled virus was collected and its titre was estimated. Desired cell line (here MCF7) was infected with 105 IU/ml virus. Infected cells were subjected to selection pressure 72 hours post-transfection. Cells were fist sorted for positive GFP signals and the GFP sorted cells were grown to establish the stable cell line. To validate the extent of knockdown, PC4 levels were checked at the protein level and compared against the nonsilencing shRNA harbouring MCF7 cells. MDA-MB-231 cells harbouring luc plasmid as well as sh-RNA against PC4 were constructed similarly as described above.

### RNA extraction from patient samples

RNA from patient samples were either obtained from paraffin-embedded tissues or from solid tumors stored in RNA Later solution. For paraffin-embedded tissues first, deparaffinization was carried out by xylene method followed by Proteinase K digestion. For solid samples, it was crushed using a sterile mortar and pestle using liquid nitrogen. The tissue homogenate was further processed by the Trizol method. To ensure efficient precipitation of the RNA the isopropanol step was done overnight. This was followed by ethanol wash and the pellet was dried similarly and dissolved in water. All the RNA extracted was analyzed through spectrophotometric analysis for absorbance at 260nm, 280 nm and 230nm. Pure RNA samples having A260/280 between 1.9-2.0 and A260/230 >1.7 were used for further gene expression analysis. The integrity of the RNA and its purity was measured by the absorbance 260/280nm, since the ratio is more than 1.5, presumably the RNA was substantially intact.

### Cloning of pre miRNAs

The pre-miR sequences of miRNA 29a, 29b and 29c were cloned into pSUPER vector. The pSUPER RNAi system provides a mammalian expression vector that guides the intracellular synthesis of mature miRNAs from the precursor. The vector uses the polymerase-III H1-RNA gene promoter, as it produces a small RNA transcript lacking a polyadenosine tail and has a well-defined start of transcription and a termination signal consisting of five thymidines in a row (T5). Most important, the cleavage of the transcript at the termination site is after the second uridine, yielding a transcript resembling the ends of synthetic siRNAs, which also contain two 3' overhanging T or U nucleotides (nt). Forward and reverse oligonucleotide sequences of miR 29 family (Sigma) were obtained and these were then annealed. pSUPER vector was double digested with BglII and HindIII and then ligated with the respective annealed oligonucleotides. Ligation was carried out at room temperature for 8hrs. Plasmids were isolated from positive colonies obtained on LB Amp plates and they were digested with EcoRI and HindIII to confirm the clones by insert release. The clones were further confirmed by sequencing.

### miRNA cDNA synthesis

For cDNA synthesis, the NCODE VLO miRNA cDNA synthesis kit was used. The NCode™ VILO™ miRNA cDNA Synthesis Kit provides qualified reagents for the tailing of microRNAs (miRNAs) and other small RNAs in a total RNA population, synthesis of the first-strand cDNA from the tailed RNA, and subsequent detection in real-time quantitative PCR (qPCR). These kits have been optimized for the detection and quantification of miRNA from 100 pg to 1 μg of total RNA, with the amount of starting material ranging as low as 10 pg. cDNAs were made from 400ng-1μg of RNA as per instruction in the manual at 37ºC for 1hour followed by heat inactivation at 90ºC for 10 minutes. cDNAs were stored at -20ºC for long time storage.

### Cell culture

MCF7 (mammary gland carcinoma cells isolated from metastatic site: pleural effusion) and HEK293 (human embryonic kidney) cells were procured from American Type Culture Collection (ATCC). ZR751 cells, a human breast carcinoma cell line derived from the derived from metastatic site: ascites, was a kind gift from Dr Amit Dutt (ACTREC, Mumbai, India). ZR751 cells were grown in RPMI-1640 media, MCF7 cells were grown in Minimum Essential Media (MEM) and HEK293 cells in Dulbecco's Modified Eagle's Media. Each of these media was supplemented with 2 mM glutamine, antibiotic solution (penicillin, streptomycin, amphotericin) and 10% fetal bovine serum (FBS). The cell stocks were stored in liquid nitrogen. The growth medium for MCF7 cells was also supplemented with 0.01 mg/ml human recombinant insulin in addition to the basic media requirements.

### Real-Time PCR analysis

1μl of respective cDNA was used in PCR reaction mix containing specific gene primers and 2X Syber Green mix (KAPA Biosystems). Sybr Green mix contains SYBR green I dye and required PCR reagents like dNTPs, DNA polymerase and compatible buffers. The first-strand cDNA synthesized was used for Real-Time/ quantitative PCR (RT-PCR/ qPCR). To the 2X SYBR Green master mix, specific primers (forward and reverse), cDNA and high ROX (for normalization) were added. The reaction was carried out in the StepOnePlus Real-time PCR system. The data was analysed in StepOne software v2.3. PCR conditions were standardised for each set of gene primers used. Fold expression change was calculated using ΔΔCt method using either Actin or U6SnRNA (in case of miRNAs) gene primers for normalization. Sensitivity and specificity of the primers were ascertained by melt curve analysis. The sequences of the primers are given below. For miRNAs universal Reverse primer from the NCODE VLO cDNA synthesis kit (Invitrogen) was used. Primers used are given as follows: hsa-miR-29a: ACCATCTGAAATCGGTTAAAA, hsa-miR-29b: CACCATTTGAAATCAGTGTTAAA, has-miR-29c: CACCATTTGAAATCGGTTAA, PC4 Fp: AGGTGAGACTTCGAGAGCCCTGT, PC4 Rp: TTCAGCTGGCTCCATTGTTCTGG, Actin Fp: AGATGTGGATCAGCAAGCAGGAGT, Actin Rp: TCCTCGGCCACATTGTGAACTTTG, U6SnRNA: GGAACGCTTCACGAATTAA.

### Luciferase assay

Human Embryonic Kidney cells (HEK 293) and also MCF7 were maintained at conditions as mentioned previously. Before transfection cells were seeded at 0.6–1*10^6 cells in 30-mm-diameter dishes. pMIR REPORT luciferase construct is used. The 3' UTR of the luciferase gene contains a multiple cloning site for insertion of predicted miRNA binding targets or other nucleotide sequences. By cloning a predicted miRNA target sequence into pMIR-REPORT, the luciferase reporter is subjected to regulation that mimics the miRNA target. The 3'UTR region of PC4 is cloned into the MCS region pMIR-REPORT luciferase vector. This luciferase vector was co-transfected with miRNA expression vectors using lipofectamine (Invitrogen).The pMIR-β-gal construct was used as an internal control. Empty vector (pSUPER) was used as a control. Prior to transfection, the medium was replaced with fresh DMEM without FBS. The constructs and Lipofectamine were incubated for a period of 20mins to ensure Lipofectamine-DNA complex formation, as per the manufacturer's protocol. After 6hrs the medium was replaced by 10% FBS supplemented DMEM medium. Luciferase and β-galactosidase activities were measured 48h after the transfection with luciferase assay and β-galactosidase assay systems according to the procedure provided by the manufacturer.

### Matrigel invasion assay

Breast cancer non-silencing control cells, as well as PC4 knockdown cells, were seeded onto BD BioCoat Matrigel matrix (Corning Life Sciences, Tewksbury, MA, USA) in the upper chamber of a 24-well culture plate at a confluency of 20,000 to 25,000 cells. The lower chamber containing the respective medium was supplemented with 10% serum as a chemoattractant. After 12-16 hours, the non-invading cells and Matrigel matrix were gently removed with a cotton swab. Invasive cells located on the lower side of the chamber were stained with 0.2% crystal violet in methanol, air-dried and photographed using an inverted microscope (× 4). For migration assays, a similar protocol was followed without the coating of the basement matrix. For quantitation at least 3 independent fields from each biological replicate were considered.

### *In vivo* tumorigenic assay

Female Nu/Nu mice weighing were obtained for use in a protocol for in-vivo experiments approved by the Animal Care and Use Committee (ACUC), Florida A & M University. The animals were acclimated to laboratory conditions for 1 week prior to experiments and were maintained on standard animal chow and water ad libitum. The room temperature was maintained at 22 ± 1 °C and the relative humidity of the experimentation room was kept in the range of 35–50%. Animals were randomized and grouped according to treatment. Stable PC4 knockdown MDAM231-Luc cells were generated and maintained in DMEM media. Sub confluent cells were harvested in 0.2 mL of PBS and injected into the mammary fat pads of each nu/nu mice (10^7^ per mouse). 6 mice in each group were taken and the tumour formation was examined weekly after 4 weeks of injections. The growth of tumours, as well as the metastatic process, was analysed using optical imaging with bioluminescence (BLI).

### Statistical analysis

All values are expressed as the mean ± S.E.M. Graphs were plotted in GraphPad PrismTM. For the statistical analysis, results were analysed using unpaired/paired t test and differences were considered significant if p< 0.01. All experiments were done in triplicates with a biological replicate represented as n.

## 















## Abbreviations

EMT: Epithelial to mesenchymal transition
HMG: High mobility group
HP1: Heterochromatin protein 1
IDC: invasive ductal carcinoma
IgG: Immunoglobulin G
IHC: Immunohistochemistry
KAT: Lysine (K) Acetyltransferase
miR: microRNA
MMP: Matrix Metalloproteinase
pri-miRNA: primary miRNA
shRNA: Short hairpin RNA
