# Supplementary material for: Chromatin protein PC4 is downregulated in breast cancer to promote disease progression: Implications of miR-29a
Source: Oncotarget. 2019 Dec 3;10(64):6855–69. doi: 10.18632/oncotarget.27325 (PMC6901337; doi:10.18632/oncotarget.27325)
Supplement: Supplementary file 1 [file oncotarget-10-6855-s001.pdf]

Chromatin protein PC4 is downregulated in breast cancer to promote disease progression: Implications of miR-29a

SUPPLEMENTARY MATERIALS

Gene expression analysis from TCGA

The patient's clinical data for breast carcinoma were downloaded using TCGA assembler using GDC server. Downloaded data comprised of tumor pathologic and node pathologic information. The information obtained is summarized in Table no.4. TCGA RNA-seq data (including raw_read_count and scaled_estimate for each sample) for all primary tumor, metastatic tumor and matched normal samples were downloaded using TCGA assembler. Transcript per million values for each gene was obtained by multiplying scaled_estimate by 1 000 000. Boxplot was generated using R (https://cran.r-project.org/).Gene expression correlation analysis of PC4, and miR-29a/b/c using R programming language.

Prediction of miRNAs targeting PC4 3'UTR

The 3Kb long 3'UTR was analyzed to determine the presence of miRNA binding sites with the help of online prediction tools such as miRANDA and TargetScan miRANDA (microRNA.org) follows a three step analysis to predict miRNAs. For the PC4 3'UTR analysis the search for miRNAs was done giving the SUB1 Huaman 3'UTR as the input. miRNA sequences from the database input are scanned against user-provided 3′ UTRs to check for Watson Crick matches. The free energy of each miRNA:mRNA target pair that exceeds a threshold matching score is calculated. Each target that has a predicted free energy below a threshold is then passed to the last step. Finally, conservation is used as a final filter. However, miRanda considers conservation of both binding site and position. Unlike most miRNA target predictors, miRanda considers matching along the entire miRNA sequence (Enright et al., 2003). It takes the seed region into account by weighting matches in the seed region more heavily. Matches are allowed to contain limited G-U wobble pairs and insertions or deletions (indels). Free energy is calculated by predicting the folding of the miRNA:mRNA hybrid using the Vienna package (Hofacker et al., 1994). Although this is a common method, it ignores any additional protein interaction, such as with the RNA-induced silencing complex (Enright et al., 2003). Based on these analysis miRSVR score is obtained based on the seed-site pairing, site context, free-energy, and conservation. A mirSVR cutoff of <= -1.2 is recommended to predict miRNAs.

TargetScan (Lewis et al., 2005; Grimson et al., 2007; Friedman et al., 2009; Garcia et al., 2011) ia another online prediction tool which allows the user to search by miRNA name, gene name, or from broadly conserved, conserved, or poorly conserved miRNA families across several species. The output screen ranks predicted targets by either the predicted efficacy of targeting (context+ scores) or the probability of conserved targeting (PCT). For conservation, the conservation of a 3′ UTR is first determined followed by analysis of a specific k-mer (8mer, 7mer-m8, or 7mer-1A). Since one 3′ UTR can contain multiple target sites, an aggregate PCT is provided. For each type of k-mer, the number is provided for that target and whether or not it is considered a conserved site or a poorly conserved site. Furthermore, there is a link to the 3′ UTR of the gene that demonstrates the conserved seed sequence (Friedman et al., 2009). The context+ score demonstrates the probability of a given target as being effectively targeted. Scoring for this feature was derived from experimental results. Several features are included when defining the score, such as 3′ compensatory pairing, local AU content, and position contribution (Grimson et al., 2007; Garcia et al., 2011).
