# Supplementary material for: Chromatin protein PC4 is downregulated in breast cancer to promote disease progression: Implications of miR-29a
Source: Oncotarget. 2019 Dec 3;10(64):6855–69. doi: 10.18632/oncotarget.27325 (PMC6901337; doi:10.18632/oncotarget.27325)
Supplement: Supplementary file 2 [file oncotarget-10-6855-s002.pdf]

Chromatin protein PC4 is downregulated in breast cancer to promote disease progression: Implications of miR-29a

SUPPLEMENTARY MATERIALS FIGURE

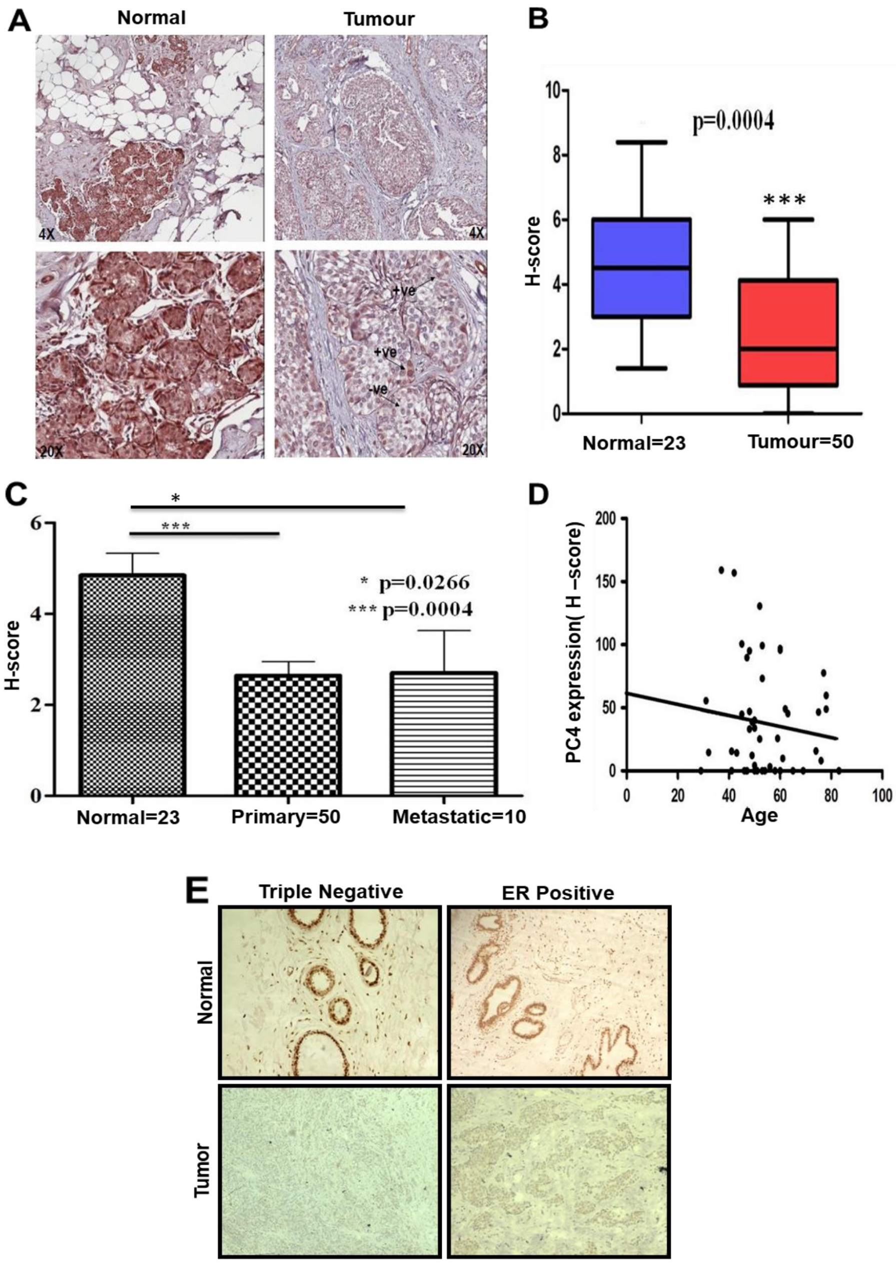

Supplementary Figure 1: PC4 expression analysis in breast cancer patient samples. **(A)** IHC analysis performed on breast cancer tissue microarrays (IMH-364, IMH-371; Imgenex) with anti PC4 antibodies. These samples belong to Moffitt Cancer Center and Research Institute, Florida, USA. **(B)** Mann Whitney test plot depicting mean H-score of the total examined normal and tumor tissues belonging to western samples. **(C)** H-Scores of normal, primary and metastatic tumor tissues of western samples. Statistical analysis has been performed by Mann- Whitney U test (Mean±SEM). *p=0.0266, ***p= 0.0004. **(D)** A line point graph showing downregulation of expression of PC4 with progression of stage of breast cancer in western population. **(E)** Representative images of IHC analysis with anti PC4 antibodies done on triple negative and ER positive tumor samples and compared with normal samples.
