# Supplementary material for: Chromatin protein PC4 is downregulated in breast cancer to promote disease progression: Implications of miR-29a
Source: Oncotarget. 2019 Dec 3;10(64):6855–69. doi: 10.18632/oncotarget.27325 (PMC6901337; doi:10.18632/oncotarget.27325)
Supplement: Supplementary file 3 [file oncotarget-10-6855-s003.pdf]

Chromatin protein PC4 is downregulated in breast cancer to promote disease progression: Implications of miR-29a

SUPPLEMENTARY MATERIALS FIGURE

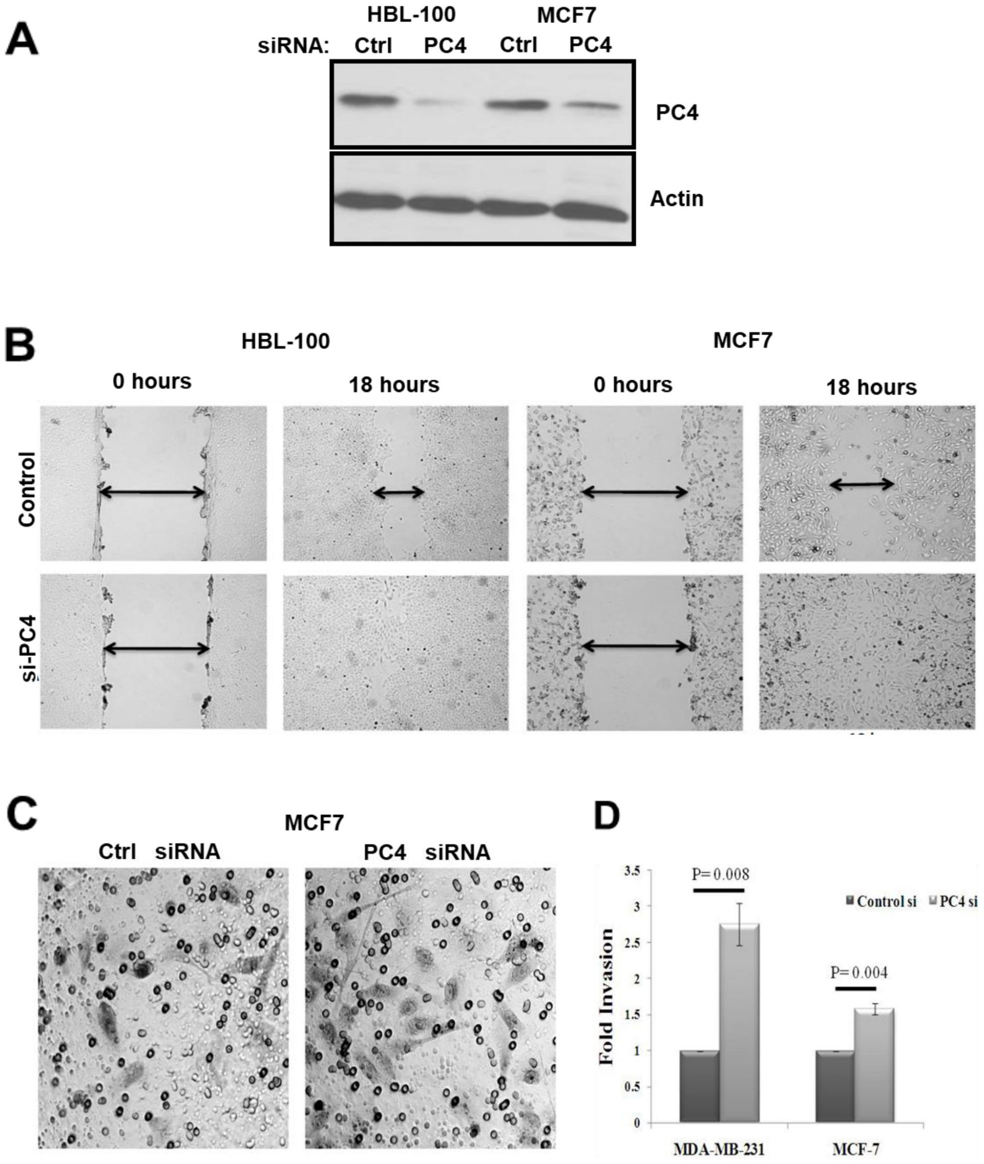

Supplementary Figure 2: Transient silencing of PC4 enhances migratory and invasive ability in breast cancer cell lines. (A) Immunoblotting analysis using anti PC4 antibodies to confirm silencing in PC4 siRNAs transfected breast cancer cell lines HBL-100 and MCF-7. Blot was reprobed with anti actin antibodies to ensure equal loading. **(B)** Wound healing assay performed in the presence of mitomycin-C in HBL-100 and MCF-7 breast cancer cell lines prior transfected with scambled (contrl si) and PC4 targetting siRNAs (PC4 si). Healing potentials were captured 18 hours post transfection. The image is representative of three independent experiments. **(C)** Boyden chamber invasion assay performed in above mentioned and MCF-7 cells. **(D)** Graph depicting fold invasion in these cell lines upon PC4 silencing.
