# Supplementary material for: Chromatin protein PC4 is downregulated in breast cancer to promote disease progression: Implications of miR-29a
Source: Oncotarget. 2019 Dec 3;10(64):6855–69. doi: 10.18632/oncotarget.27325 (PMC6901337; doi:10.18632/oncotarget.27325)
Supplement: Supplementary file 5 [file oncotarget-10-6855-s005.pdf]

Chromatin protein PC4 is downregulated in breast cancer to promote disease progression: Implications of miR-29a

SUPPLEMENTARY MATERIALS TABLE

Supplementary Table 1: Summary of the patient sample data obtained from TCGA database. N represents the number of patient samples.

TCGA–Breast cancer transcriptome (BRCA) cohort		1090	
**TCGA-BRCA cohort with PC4 and miR-29 expression data available**		1069	
**BRCA subtype**	**N**	**Tumour Stage**	**N**
Luminal A	388	I	179
Luminal B	321	II	606
Her2	111	III	242
Basal	180	IV	20
Normal-like	69	NA	22
Total	1069	Total	1069
