# Supplementary material for: Chromatin protein PC4 is downregulated in breast cancer to promote disease progression: Implications of miR-29a
Source: Oncotarget. 2019 Dec 3;10(64):6855–69. doi: 10.18632/oncotarget.27325 (PMC6901337; doi:10.18632/oncotarget.27325)
Supplement: Supplementary file 6 [file oncotarget-10-6855-s006.pdf]

Chromatin protein PC4 is downregulated in breast cancer to promote disease progression: Implications of miR-29a

SUPPLEMENTARY MATERIALS TABLE

Supplementary Table 2: Prediction of PC4 3’UTR through miRANDA. miR29 family showed higher conservation and perfect Watson crick pairing, resulting in good miRSVR score

<table>
<tr><td>Position 1977-1983 of SUB1 3' UTR</td><td>5' ...CAAUCUACUAACAGAUGGUGCUG...</td><td>mirSVR score</td></tr>
<tr><td></td><td>||||||||</td><td>-0.4186</td></tr>
<tr><td>hsa-miR-29c</td><td>3' AUUGGCUAAAGUUUACCACGAU</td><td></td></tr>
<tr><td>Position 1977-1983 of SUB1 3' UTR</td><td>5' ...CAAUCUACUAACAGAUGGUGCUG...</td><td></td></tr>
<tr><td></td><td>|||||||||||</td><td>-0.4186</td></tr>
<tr><td>hsa-miR-29a</td><td>3' AUUGGCUAAAGUCUACCACGAU</td><td></td></tr>
<tr><td>Position 1977-1983 of SUB1 3' UTR</td><td>5' ...CAAUCUACUAACAGAUGGUGCUG...</td><td></td></tr>
<tr><td></td><td>|||||||</td><td>-0.4186</td></tr>
<tr><td>hsa-miR-29b</td><td>3' UUGUGACUAAAGUUUACCACGAU</td><td></td></tr>
</table>
