# Supplementary material for: Chromatin protein PC4 is downregulated in breast cancer to promote disease progression: Implications of miR-29a
Source: Oncotarget. 2019 Dec 3;10(64):6855–69. doi: 10.18632/oncotarget.27325 (PMC6901337; doi:10.18632/oncotarget.27325)
Supplement: Supplementary file 7 [file oncotarget-10-6855-s007.pdf]

Chromatin protein PC4 is downregulated in breast cancer to promote disease progression: Implications of miR-29a

SUPPLEMENTARY MATERIALS TABLE

Supplementary Table 3: Molecular characteristics of the breast cancer cell lines harbouring low PC4 levels. The table summa rizes the origin and receptor status of the cell lines which express low levels of PC4 both at transcript as well as protein level

Cell line	Subtype#	ER*	PR*	ERBB2/ HER2*	Source	Tumor Type
HCC38	Basal B	-	-	-	primary tumor	ductal carcinoma
HCC1806	NA	-	-	-	primary tumor	Squamous Carcinoma
SKBR3	Luminal	-	-	+	pleural effusion	adenocarcinoma
ZR751	Luminal	+	-	-	ascites fluid	Invasive ductal carcinoma
