# Supplementary material for: Chromatin protein PC4 is downregulated in breast cancer to promote disease progression: Implications of miR-29a
Source: Oncotarget. 2019 Dec 3;10(64):6855–69. doi: 10.18632/oncotarget.27325 (PMC6901337; doi:10.18632/oncotarget.27325)
Supplement: Supplementary file 4 [file oncotarget-10-6855-s004.pdf]

Chromatin protein PC4 is downregulated in breast cancer to promote disease progression: Implications of miR-29a

SUPPLEMENTARY MATERIALS FIGURE

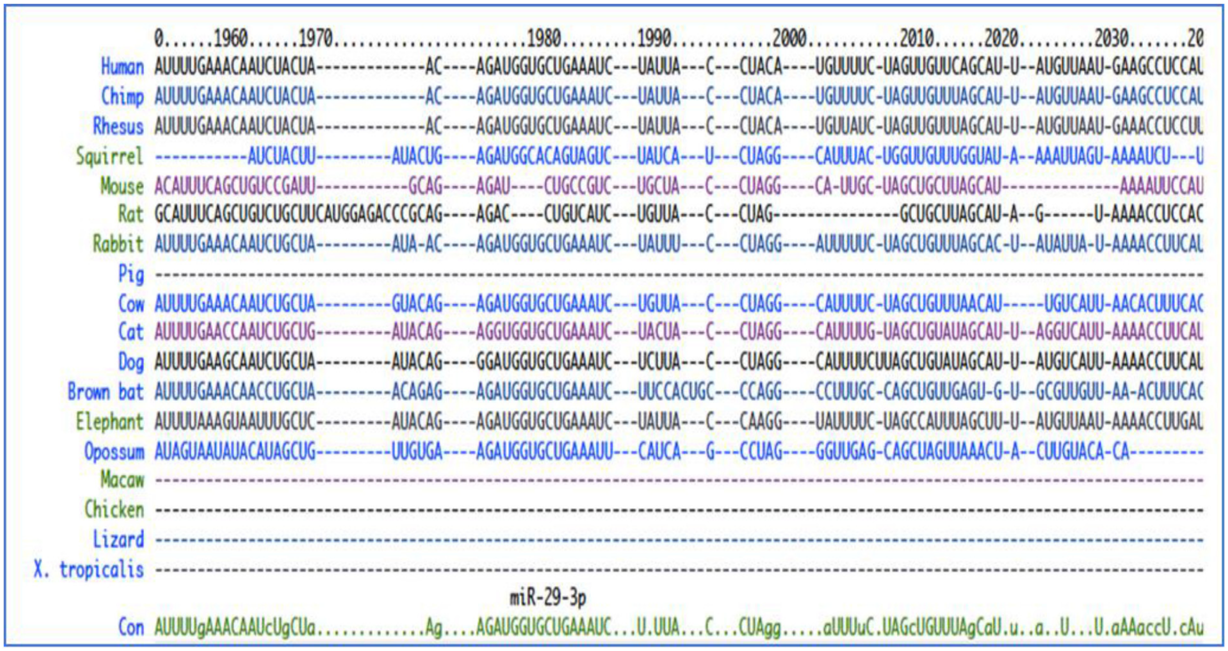

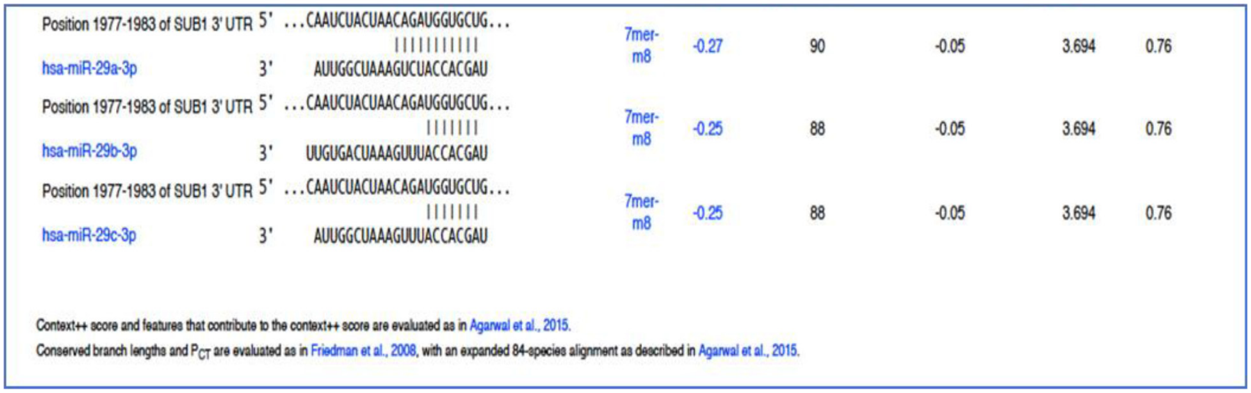

		Predicted consequential pairing of target region (top) And miRNA (bottom)	Site type	Context +++ score	Context +++ score percentile	Weighted Context +++ score	Conserved Branch length	P_{CT}
Position 1977-1983 of SUB1 3' UTR / hsa-miR-29a-3p	5' / 3'	...CAAUCUACUAACAGAUGGUGCUG... |||||||||| AUUGGCUAAAGUCUACCACGAU	7mer-m8	-0.27	90	-0.05	3.694	0.76
Position 1977-1983 of SUB1 3' UTR / hsa-miR-29b-3p	5' / 3'	...CAAUCUACUAACAGAUGGUGCUG... |||||| UUGUGACUAAAGUUUACCACGAU	7mer-m8	-0.25	88	-0.05	3.694	0.76
Position 1977-1983 of SUB1 3' UTR / hsa-miR-29c-3p	5' / 3'	...CAAUCUACUAACAGAUGGUGCUG... |||||| AUUGGCUAAAGUUUACCACGAU	7mer-m8	-0.25	88	-0.05	3.694	0.76

Context++ score and features that contribute to the context++ score are evaluated as in Agarwal et al., 2015.
Conserved branch lengths and P_{CT} are evaluated as in Friedman et al., 2008, with an expanded 84-species alignment as described in Agarwal et al., 2015.

Supplementary Figure 3: Analysis of miR29 binding. Alignment of the miR29 binding site with PC4 3'UTR of different species to determine the conservation (Upper panel). Scoring of the binding of miR29a, 29b, 29c to the 3'UTR of PC4 based on the seed sequence matching and conservation (PCT value) (Downloaded from TargetScan website).
